# NapR Regulates the Expression of Phosphoserine Aminotransferase SerC to Modulate Biofilm Formation and Resistance to Serine Stress of Mycobacteria

**DOI:** 10.3390/ijms26052181

**Published:** 2025-02-28

**Authors:** Minhao Guo, Xiaocui Ling, Linzhao He, Yukuo Gou, Zhun Li, Weihui Li

**Affiliations:** State Key Laboratory for Conservation and Utilization of Subtropical Agro-Bioresources, College of Life Science and Technology, Guangxi University, Nanning 530004, China

**Keywords:** transcription factor, phosphoserine aminotransferase, mycobacteria, biofilm formation, serine stress

## Abstract

*Mycobacterium tuberculosis* is a formidable pathogen capable of establishing persistent infections within macrophages. To survive and thrive within the host environment, it has evolved intricate regulatory networks, including a diverse array of transcription factors that enable adaptation to various stresses encountered within the host. However, the mechanisms by which transcription factors regulate biofilm formation in *M. tuberculosis* remain incompletely understood. This study aimed to investigate the role of *serC*, encoding phosphoserine aminotransferase, and its regulation by NapR, a transcription factor, in mycobacterial physiology. NapR regulates *serC* through directly binding to its promoter. Notably, the regulatory effect and corresponding phenotypes vary due to distinct binding affinities of NapR for the *serC* promoter in different mycobacterial species. In *Mycobacterium smegmatis*, NapR_Msm_ positively regulates biofilm formation, growth on solid media, and the transition from microcolonies to microcolonies by activating *serC_Msm_*. In the *BCG vaccine*, on the contrary, NapR_BCG_ represses *serC_BCG_*, thus negatively regulating colony size and alleviating the growth inhibition caused by high concentrations of serine. Furthermore, proteomic analysis suggested NapR serves as a global transcriptional regulator in *BCG vaccine* strains by simultaneously modulating four metabolic pathways. These findings underscore the complex and strain-specific regulatory mechanisms governing serine metabolism in mycobacteria and provide valuable insights into the interplay between metabolism, gene regulation, and bacterial physiology.

## 1. Introduction

Pulmonary tuberculosis is a zoonotic infectious disease caused by infection with the *Mycobacterium tuberculosis* complex (MTB). In 2023, an estimated 400,000 cases of MDR-TB were found [1]. The *BCG vaccine*, a live attenuated strain of *Mycobacterium bovis*, is administered to children for the prevention of tuberculosis clinically [2]. *M. tuberculosis* is capable of infecting macrophages and inhibiting the maturation of phagolysosomes [3], thereby ensuring long-term intracellular survival within macrophages [4]. To survive within this hostile environment, *M. tuberculosis* encounters various stressors, including but not limited to oxidative stress, reductive stress, acid stress, and nutrient deprivation. To promptly respond to these unfavorable environmental challenges, *M. tuberculosis* possesses a sophisticated regulatory network, including a diverse array of transcription factors that orchestrate cellular responses to environmental cues [5].

*M. tuberculosis* is subjected to a variety of external adverse stressors, including but not limited to oxidative stress, reductive stress, acid stress, and nutrient deprivation. To promptly respond to these unfavorable environmental challenges, *M. tuberculosis* possesses an extensive array of transcription factors within its cells, ensuring a rapid and effective response to external stresses [5]. Transcription factors need to interact with RNA polymerase to exert their transcriptional regulatory activity. RNA polymerase depends on sigma factors to recognize promoters and ensure transcription specificity. Activating transcription factors enhance transcription by altering promoter structure, interacting with sigma factors, or binding the RNA polymerase α-subunit. In contrast, repressive transcription factors inhibit mRNA synthesis by blocking RNA polymerase binding, modifying promoter topology, or antagonizing activators [6]. To date, at least 35 transcription factors and 11 nucleoid-associated proteins have been identified in *M. tuberculosis* [5,7]. These molecules are extensively involved in the bacterium’s response to external stressors. For example, c-di-GMP regulates the antioxidant capacity of Mycobacterium by activating LtmA and inhibiting HpoR [8]. Whib7 mediates the resistance of Mycobacterium to aminoglycosides, β-lactams, macrolides, and quinolones [9]. However, current research has not yet clarified which transcription factors regulate biofilm formation in Mycobacterium.

Biofilms are typically defined as “a sessile community derived from microorganisms, characterized by cells that irreversibly attach to a substrate or interface, or to one another, become embedded in a self-produced extracellular polymeric matrix, and exhibit altered phenotypes with respect to growth rate and gene transcription”. Under certain environmental stimuli, planktonic (free-floating) bacteria can initiate the transition from a planktonic state to a complex, surface-attached community structure [10]. Biofilms are composed of biofilm cells and a matrix of hydrated extracellular polymeric substances (EPSs), with EPSs accounting for over 90% of the biofilm’s dry weight [11]. Extensive in vitro studies have shown that biofilms contribute to the development of drug resistance in *Mycobacterium tuberculosis*. Even rifampin-sensitive mutant strains can regain resistance to rifampin when grown within biofilms [12]. In *M. tuberculosis* infections, biofilm formation has been implicated in the establishment of persistent infections. Studies have demonstrated the presence of cellulose-dominant biofilms in the lung tissues of tuberculosis patients, contributing to the development of drug tolerance [13]. However, the mechanism of regulating biofilm formation in *M. tuberculosis* remains incompletely understood. Recent studies have highlighted the crucial role of nucleoid-associated proteins in biofilm formation. Lsr2 has been shown to mediate biofilm development induced by high concentrations of c-di-GMP through transcriptional upregulation of *hadD* [14].

Serine, a non-essential amino acid, plays a pivotal role in various cellular processes, including protein synthesis, one-carbon metabolism, and lipid biosynthesis. Serine de novo synthesis originates from glycolysis, where 3-phosphoglycerate (3-PG) is sequentially oxidized by SerA to form 3-phosphohydroxypyruvate (3-PHP), converted to 3-phosphoserine (3-PS) by SerC with glutamate deamination, and dephosphorylated by SerB to produce L-serine [15]. Accordingly, SerC is necessary for serine metabolism and essential for mycobacterial growth and survival. A study on *M. smegmatis* demonstrated that *serC* expression is induced in response to various environmental stresses, including oxidative stress, nitric oxide (NO), membrane-denaturing agents, and antibiotic exposure [16]. Isoniazid, rifampin, and H_2_O_2_ induce the upregulation of *serC* expression in *M. smegmatis*, while inhibiting *serC* expression enhances the bactericidal effect of these agents and H_2_O_2_ on *M. smegmatis* [16].

In our previous screening, we identified a *M. smegmatis* strain exhibiting a smooth colony surface morphology. Subsequent sequencing revealed that the expression of the intracellular *serC* was suppressed in this strain. Here, we demonstrate that *serC*, a key enzyme in serine biosynthesis, plays a crucial role in various aspects of mycobacterial physiology, including growth, biofilm formation, and adaptation to environmental stresses. Furthermore, we elucidate the contrasting regulatory roles of a novel transcription factor NapR on *serC* in *M. smegmatis* and the *BCG vaccine* strains, highlighting the complex and strain-specific nature of these regulatory networks.

## 2. Results

### 2.1. serC_Msm_ Positively Regulates Biofilm Formation of M. smegmatis

**Figure 1:**
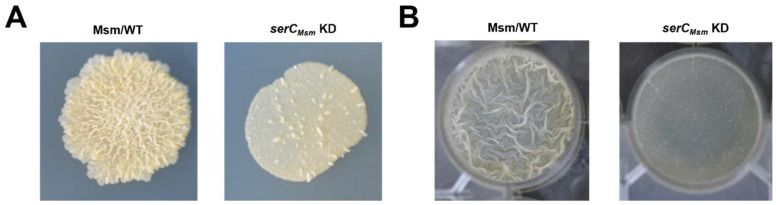
Effects of *serC_Msm_* repression on the physiological phenotypes of *M. smegmatis*. (**A**) Spot colony morphology of the Msm/WT strain and the *serC_Msm_* KD strain in the presence of anhydrotetracycline. (**B**) The biofilm formation at the air–liquid surface of the Msm/WT strain and the *serC_Msm_* KD strain in the presence of anhydrotetracycline.

In our preliminary experiment, we developed a whole-genome CRISPR interference (CRISPRi) library in *M. smegmatis* that produced colonies with a rough surface morphology on 7H10 solid medium (Figure S1A) [17]. Upon treatment with anhydrotetracycline to activate the CRISPRi system, an *M. smegmatis* strain with a smooth colony surface was screened out (Figure 1A). Subsequent sequencing analysis indicated that the suppression of the *serC_Msm_* gene was responsible for this phenotype. Next, the biofilm formation capability at the air–liquid interface was investigated in the *serC_Msm_*-repressed strain (*serC_Msm_* KD). In the absence of anhydrotetracycline, biofilm formation in the *serC_Msm_* KD strain was comparable to that of the wild-type *M. smegmatis* (Msm/WT) (Figure S1B). However, when anhydrotetracycline was present, the *serC_Msm_* KD strain exhibited significantly impaired biofilm formation relative to the Msm/WT strain (Figure 1B).

### 2.2. NapR_Msm_ Directly Binds to the serC_Msm_ Promoter and Activates serC_Msm_

To further elucidate the regulatory mechanisms governing *serC_Msm_* expression, we utilized the TB Database to search for potential transcription factors of its homologous gene *serC_Mtb_* in *M. tuberculosis*. This analysis revealed Rv3050c as a potential transcriptional regulator of *serC_Mtb_*, which we named NapR_Mtb_. Subsequently, we investigated the potential regulatory role of NapR_Msm_ (encoded by *msmeg_1029*), the *M. smegmatis* homolog of NapR_Mtb_, on *serC_Msm_* expression.

**Figure 2:**
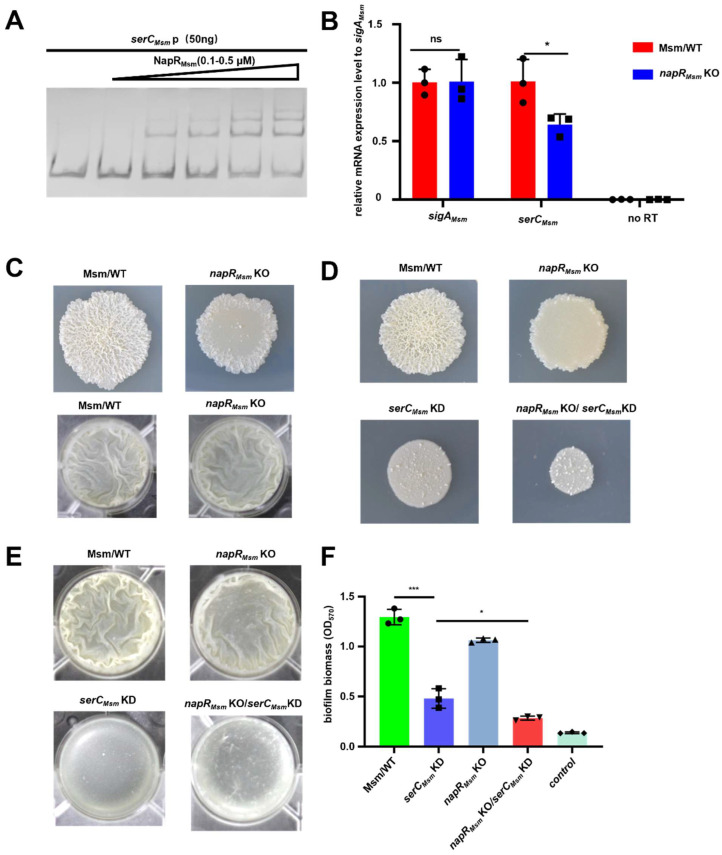
NapR_Msm_ activates *serC_Msm_* to positively regulate biofilm formation in *M. smegmatis*. (**A**) EMSA assays for the *serC_Msm_* promoter DNA-binding activity of NapR_Msm_. *serC_Msm_*p was co-incubated with increasing amounts of NapR_Msm_. (**B**) RT-qPCR analysis for mRNA levels of *serC_Msm_* in Msm/WT and *napR_Msm_* KO strains. *sigA_Msm_* was used as a reference control. (**C**) Spot colony morphology and biofilm formation at the air–liquid surface of the Msm/WT strain and the *napR_Msm_* KO strain. (**D**) Spot colony morphology of the Msm/WT strain, *napR_Msm_* KO strain, *serC_Msm_* KD strain, and the *napR_Msm_* KO/*serC_Msm_* KD strain under anhydrotetracycline induction. (**E**) The biofilm formation at the air–liquid surface of the Msm/WT strain, *napR_Msm_* KO strain, *serC_Msm_* KD strain, and the *napR_Msm_* KO/*serC_Msm_* KD strain under anhydrotetracycline induction. (**F**) Quantitation of biofilm biomass by crystal violet staining. ns *p* ≥ 0.05, * *p* < 0.05, *** *p* < 0.001.

First, we performed an electrophoretic mobility shift assay (EMSA) to examine the in vitro binding capacity of NapR_Msm_ to the *serC_Msm_* promoter (*serC_Msm_* p). The results showed that NapR_Msm_ binds directly to *serC_Msm_* p (Figure 2A). To further validate the transcriptional regulatory role of NapR_Msm_ on *serC_Msm_* expression, we constructed an *M. smegmatis* mutant lacking *napR_Msm_* (*napR_Msm_* KO). An RT-qPCR assay revealed a significant downregulation of *serC_Msm_* mRNA level in the *napR_Msm_* KO strain compared to Msm/WT (Figure 2B). These findings collectively demonstrate that NapR_Msm_ functions as a transcriptional activator of *serC_Msm_* by directly binding to its promoter.

### 2.3. NapR_Msm_ Activates serC to Positively Regulate Biofilm Formation in M. smegmatis

Considering that NapR_Msm_ works as transcriptional activator of *serC_Msm_*, and *serC_Msm_* plays a crucial role in *M. smegmatis* biofilm formation, we investigated the impact of NapR_Msm_ on the colony surface morphology and biofilm formation capability at the air–liquid interface in *M. smegmatis*. Consistent with the *serC_Msm_* KD strain, the *napR_Msm_* KO strain exhibited a smooth colony surface and impaired biofilm formation compared to the Msm/WT strain (Figure 2C). To further dissect the contribution of NapR_Msm_-mediated *serC_Msm_* activation to these phenotypes, we generated an *M. smegmatis* strain lacking *napR_Msm_* with simultaneous repression of *serC_Msm_* expression (*napR_Msm_* KO/*serC_Msm_* KD). The results indicated that the *napR_Msm_* KO/*serC_Msm_* KD strain exhibited the most pronounced smooth and smallest colony morphology, as well as the most severe biofilm deficiency, compared to the Msm/WT, *serC_Msm_* KD, and *napR_Msm_* KO strains (Figure 2D,E). Notably, no significant differences were observed in the control group without anhydrotetracycline induction (Figure S2A,B). Additionally, quantitative biofilm formation assays using crystal violet staining corroborated these findings, with the *napR_Msm_* KO/*serC_Msm_* KD strain displaying the most significant biofilm reduction, followed by the *napR_Msm_* KO and *serC_Msm_* KD strains (Figure 2F and Figure S2C).

In summary, *serC_Msm_* positively regulates colony radius and biofilm formation in *M. smegmatis*. The absence of *napR_Msm_* in the *serC_Msm_* KD strain further exacerbates the reduction in colony radius and biofilm formation. These findings demonstrate that NapR_Msm_ positively regulates colony radius and biofilm formation in *M. smegmatis* by activating *serC_Msm_*.

### 2.4. NapR_Msm_ Activates serC_Msm_ and Influences the Growth State of M. smegmatis on Solid Surfaces

It was observed that the colony radius of the *serC_Msm_* KD and *napR_Msm_* KO strains was smaller than that of Msm/WT (Figure 2D). Given that *serC_Msm_* is involved in serine biosynthesis and cellular growth, we investigated the impact of *serC_Msm_* and *napR_Msm_* on *M. smegmatis* growth. Growth curves of the Msm/WT, *napR_Msm_* KO, *serC_Msm_* KD, and *napR_Msm_* KO/*serC_Msm_* KD strains were determined in 7H9 liquid medium containing 0 mM or 5 mM serine under shaking conditions (37 °C, 160 rpm). Surprisingly, only the *serC_Msm_* KD and *napR_Msm_* KO/*serC_Msm_* KD strains showed slight growth inhibition, which was reversed by the addition of serine (Figure S3A). Moreover, no significant growth differences were observed among these strains under static growth conditions in 7H9 liquid medium at 37 °C (Figure S3B). These findings suggest that *serC_Msm_* and *napR_Msm_* do not significantly affect the growth of *M. smegmatis* in liquid media, and the observed biofilm formation defects due to *serC_Msm_* repression or *napR_Msm_* knockout are not attributed to growth inhibition.

**Figure 3:**
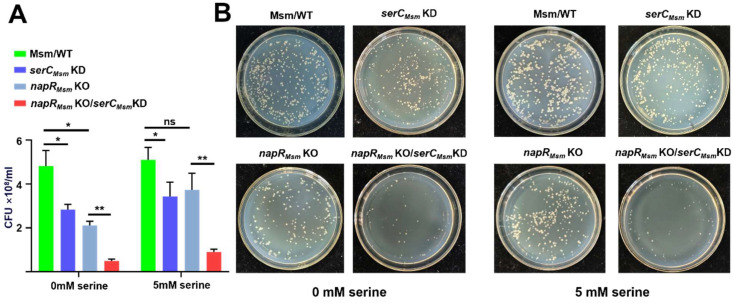
NapR_Msm_ activates *serC_Msm_* to positively regulate the growth of *M. smegmatis* on solid media. (**A**) Quantification of colony counts of *M. smegmatis* on 7H10 solid medium. (**B**) Representative images of *M. smegmatis* colonies on 7H10 solid medium. Equal amounts of the Msm/WT, *serC_Msm_* KD, *napR_Msm_* KO and *napR_Msm_* KO/*serC_Msm_* KD strains were plated on 7H10 solid medium supplemented with 0 mM or 5 mM serine, and colony counts were recorded after 4 days. ns *p* ≥ 0.05, * *p* < 0.05, ** *p* < 0.01.

To further investigate the impact of *serC_Msm_* on growth under solid-phase conditions, we sampled the statically cultured *M. smegmatis* strains and plated them onto 7H10 solid medium. In contrast to liquid culture, both *napR_Msm_* KO and *serC_Msm_* KD strains exhibited a significant reduction in colony-forming units (CFUs) compared to Msm/WT, while the *napR_Msm_* KO/*serC_Msm_* KD strain showed a more pronounced decrease, with approximately one-tenth the CFU count of Msm/WT (Figure 3A). Notably, the addition of serine only partially restored the growth inhibition observed on solid media, indicating that *serC_Msm_* may affect *M. smegmatis* growth on solid media through mechanisms beyond serine biosynthesis.

According to the established biofilm formation model, biofilm maturation can be subdivided into three phases: the microcolony phase, the macrocolony phase, and the mature biofilm phase [18]. Microscopic examination of colonies on 7H10 agar revealed a coexistence of microcolonies and macrocolonies. Importantly, the loss of *napR_Msm_* and reduced *serC_Msm_* expression resulted in an increased proportion of microcolonies, with the *napR_Msm_* KO/*serC_Msm_* KD strain exhibiting the highest proportion of microcolonies (Figure 3B).

Overall, *serC_Msm_* positively regulates *M. smegmatis* growth on solid media but not in liquid media, and influences the transition from microcolonies to macrocolonies during biofilm development. Furthermore, the absence of *napR_Msm_* in the *serC_Msm_* KD background exacerbates the growth inhibition on solid media and further disrupts the transition from microcolonies to macrocolonies. In conclusion, NapR_Msm_ positively regulates the colony count and the progression of biofilm development by activating *serC_Msm_*.

### 2.5. NapR_BCG_ Directly Represses the Expression of serC_BCG_

Based on sequence alignment, we found that NapR_Msm_ in *M. smegmatis* shares up to 76.6% homology with NapR_BCG_ in the *BCG vaccine* strain (Figure S4). Meanwhile, SerC_Msm_ in *M. smegmatis* shares up to 81.5% homology with SerC_BCG_ in the *BCG vaccine* strain (Figure S5). As the *BCG vaccine* is an attenuated strain of *M. bovis*, we used the *BCG vaccine* as a representative pathogenic strain to investigate how NapR_BCG_ regulates *serC_BCG_* and to explore its effects on the physiological phenotypes of the *BCG vaccine*.

**Figure 4:**
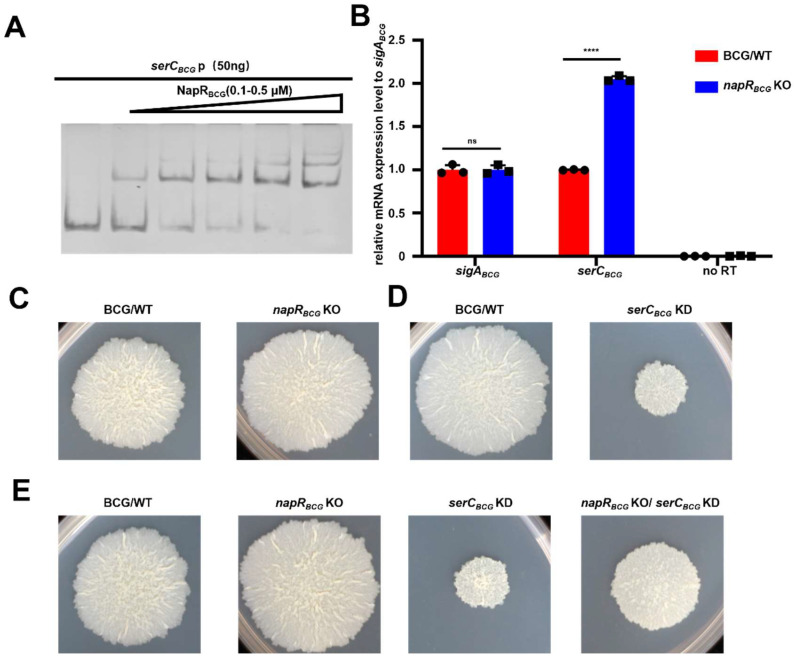
NapR_BCG_ represses *serC_BCG_* to negatively regulate biofilm formation in the *BCG vaccine* strain. (**A**) EMSA assays for the *serC_BCG_* promoter DNA-binding activity of NapR_BCG_. *serC_BCG_*p was co-incubated with increasing amounts of NapR_BCG_. (**B**) RT-qPCR analysis for the expression of levels of *serC_BCG_* in BCG/WT and *napR_BCG_* KO strains. *sigA_BCG_* was used as a reference control. (**C**) Spot colony morphology of BCG/WT and *napR_BCG_* KO strains. (**D**) Spot colony morphology of BCG/WT and *serC_BCG_* KD strains under anhydrotetracycline induction. (**E**) Spot colony morphology of the BCG/WT, *napR_BCG_* KO, *serC_BCG_* KD, and *napR_BCG_* KO/*serC_BCG_* KD strains under anhydrotetracycline induction. ns *p* ≥ 0.05, **** *p* < 0.0001.

First, we performed an EMSA to examine the in vitro binding of NapR_BCG_ to the *serC_BCG_* promoter (*serC_BCG_*p). The results showed that a concentration of 0.5 μM NapR_BCG_ was sufficient to fully saturate 50 ng of *serC_BCG_*p, indicating that there is specific binding between NapR_BCG_ and *serC_BCG_*p (Figure 4A). To verify the transcriptional regulatory relationship between NapR_BCG_ and *serC_BCG_*, we constructed a *napR_BCG_* knockout mutant (*napR_BCG_* KO) in the *BCG vaccine* strain. Surprisingly, RT-qPCR analysis using *sigA_BCG_* as an internal control revealed an upregulation of *serC_BCG_* mRNA level in the *napR_BCG_* KO strain compared to the wild-type *BCG vaccine* strain (BCG/WT) (Figure 4B). These findings demonstrate that NapR_BCG_ represses *serC_BCG_* at the transcriptional level by directly binding to its promoter, which contrasts with the activating role of NapR_Msm_ on *serC_Msm_* expression.

### 2.6. NapR_BCG_ Influences BCG Vaccine Colony Size by Repressing serC_BCG_

To investigate the effect of NapR_BCG_ on the physiological phenotype of the *BCG vaccine* strain, we examined the colony morphology of the *napR_BCG_* KO mutant. In contrast to the observations in *M. smegmatis*, the *napR_BCG_* KO strain produced colonies with an increased radius compared with the BCG/WT strain (Figure 4C). Next, we constructed a CRISPRi-based *serC_BCG_* knockdown strain (*serC_BCG_* KD), as well as a *napR_BCG_* KO/*serC_BCG_* KD double mutant. Upon induction with anhydrotetracycline, the *serC_BCG_* KD strain exhibited smaller and smoother colony morphology compared to BCG/WT (Figure 4D). However, the *napR_BCG_* KO/*serC_BCG_* KD strain reversed this change, exhibiting a larger colony size than the *serC_BCG_* KD strain, but still smaller than the *napR_BCG_* KO strain (Figure 4E). No such phenotype was observed in the absence of anhydrotetracycline (Figures S6 and S7). These findings demonstrate that *serC_BCG_* positively regulates colony size in the *BCG vaccine*, while NapR_BCG_ exerts a negative regulatory effect on *BCG vaccine* colony size by repressing *serC_BCG_*.

### 2.7. NapR_BCG_ Relieves High-Serine Stress by Repressing serC_BCG_

**Figure 5:**
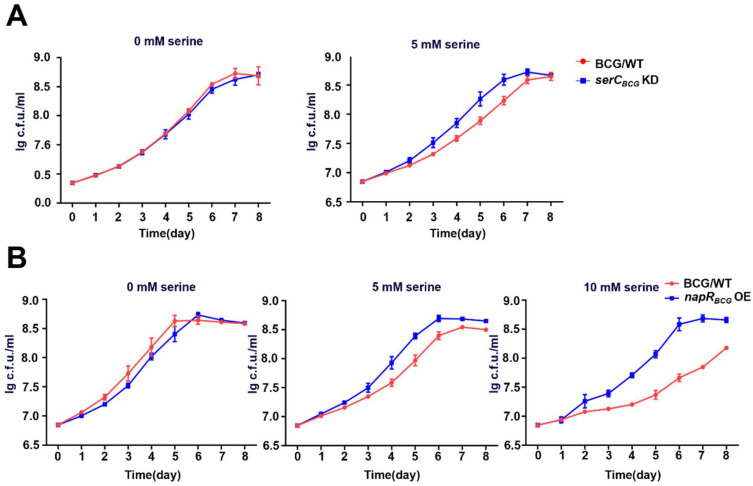
NapR_BCG_ represses the expression of *serC_BCG_* to alleviate growth inhibition caused by high serine concentrations in *BCG vaccine*. (**A**) Growth curves of BCG/WT and *serC_BCG_* KD strains under the condition of 0 mM or 5 mM serine. (**B**) Growth curves of BCG/WT and *napR_BCG_* OE strains under the condition of 0 mM, 5 mM, or 10 mM serine.

Since *serC_BCG_* encodes phosphoserine aminotransferase, and is involved in serine metabolism, we further measured the growth curves of the BCG/WT and *serC_BCG_* KD strains in 7H9 liquid medium supplemented with varying concentrations of serine (Figure 5A). In the absence of exogenous serine, BCG/WT displayed slightly better growth than *serC_BCG_* KD. However, the addition of 5 µM serine resulted in a significant growth inhibition of BCG/WT, while the *serC_BCG_* KD strain displayed a pronounced growth advantage over BCG/WT, suggesting serine supplementation induces stress for the *BCG vaccine*, and downregulation of *serC_BCG_* confers increased resistance to serine-induced stress.

To further clarify the physiological role of NapR_BCG_ under high-serine conditions, we constructed a *napR_BCG_* overexpression strain (*napR_BCG_* OE) and examined its growth under different serine concentrations (Figure 5B). Similarly, BCG/WT grew better than *napR_BCG_* OE without serine supplementation. However, as the serine concentration increased, the growth inhibition of BCG/WT intensified. In contrast, *napR_BCG_* OE almost completely overcame the inhibitory effect of high serine levels on the growth.

Collectively, the *BCG vaccine* is more susceptible to growth inhibition induced by high serine concentrations compared with *M. smegmatis*. *serC_BCG_* negatively regulates the serine stress tolerance of the *BCG vaccine*, whereas *NapR_BCG_* positively regulates the *BCG vaccine*’s tolerance to serine stress by repressing the expression of *serC_BCG_*.

### 2.8. Proteomic Analysis for the Targets of NapR_BCG_

**Figure 6:**
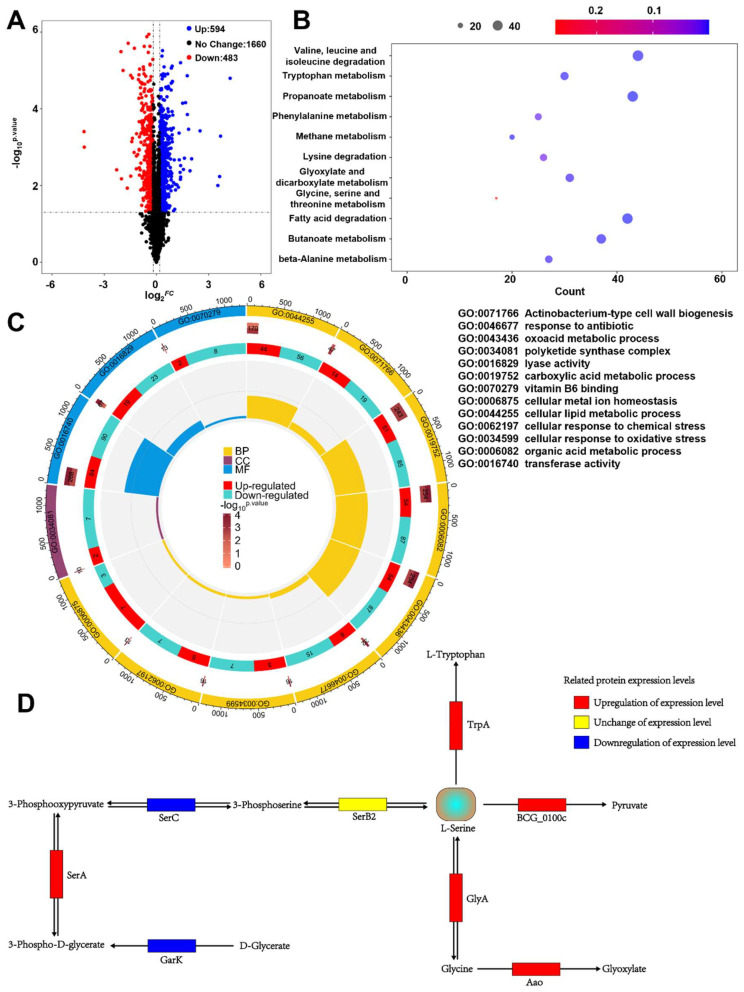
Proteomic analysis for the targets of *napR_BCG_*. (**A**) Volcano plot of the proteomic data. (**B**) KEGG enrichment analysis of the proteomic data. (**C**) GO enrichment analysis of the proteomic data. (**D**) Four metabolic pathways involved in the reduction of intracellular serine concentrations in the *napR_BCG_* OE strain.

To elucidate the specific mechanism by which *napR_BCG_* regulates serine metabolism, we conducted a proteomic analysis of the *napR_BCG_* OE strain. As expected, NapR_BCG_ expression was markedly higher in the *napR_BCG_* OE group than in the BCG/WT group (Figure S8A). Across the proteomic, a total of 2737 proteins were identified. Based on the screening criteria of *p*-value ≤ 0.05 and fold change (FC) ≥ 1.2, we identified 1077 differentially expressed proteins, suggesting that NapR_BCG_ acts as a global transcriptional regulator in the *BCG vaccine* (Figure 6A).

Subsequently, Kyoto Encyclopedia of Genes and Genomes (KEGG) (Figure 6B), and Gene Ontology (GO) (Figure 6C) enrichment analyses were performed to probe the underlying mechanisms. KEGG analysis revealed that *napR_BCG_* is a key regulator of amino acid metabolism in the *BCG vaccine*, and is involved in the regulation of valine, leucine, isoleucine, tryptophan, alanine, phenylalanine, lysine, serine, and glycine metabolism. GO enrichment analysis further indicated that NapR_BCG_ regulates the expression of 154 hydrolases and 42 transferases, contributes to the metabolism of carboxylic acids, organic acids, and oxygenated acids, and is involved in cell wall synthesis and vitamin B6 biosynthesis in the *BCG vaccine*. In addition, NapR_BCG_ plays a role in the *BCG vaccine*’s response to multiple stress factors, including antibiotics, oxidative stress, and ion homeostasis. The expression of isocitrate lyase (ICL), associated with resistance to isoniazid (INH), rifampin (RIF), and streptomycin (STREP), was upregulated 1.33-fold; catalase KatG, linked to Mycobacterium sensitivity to INH and oxidative stress, was upregulated 1.46-fold; superoxide dismutase SodA was increased 1.33-fold, and Mycobacterium ferritin BfrB 1.25-fold [19,20,21].

Based on our KEGG analysis, we identified 15 differentially expressed proteins within the glycine, serine, and threonine metabolism pathway (ko00260) (Figure 6B), with 8 proteins upregulated and 7 proteins downregulated (Figure S8B). Consistent with our findings, overexpression of *napR_BCG_* resulted in a decreased expression level of *serC_BCG_*, confirming its role as a transcriptional repressor of *serC_BCG_* (Figure S8B). To further investigate the impact of *napR_BCG_* on serine metabolism, we performed a protein–protein interaction analysis of the enriched proteins (Figure S8C). The results revealed that *serC_BCG_* serves as a central hub in the ko00260 interaction network, while TrpB and GlyA also occupy key positions.

Drawing on the protein–protein interaction analysis, we conducted an in-depth examination of the roles of these 15 proteins in the ko00260 metabolic pathway. Our findings indicate that increased *napR_BCG_* expression decreases the intracellular serine levels in the *BCG vaccine* through four distinct pathways (Figure 6D). First, upregulation of *napR_BCG_* enhances the expression of BCG_0100c and TrpA, irreversibly converting serine into pyruvate or L-tryptophan [22,23,24]. Second, increased *napR_BCG_* expression leads to upregulation of GlyA and Aao. Although GlyA reversibly converts serine into glycine, the upregulation of Aao irreversibly degrades glycine into glyoxylate, thereby driving serine flux towards glycine formation [25,26]. Finally, *napR_BCG_* represses *serC_BCG_*, blocking the conversion of 3-phosphohydroxypyruvate into serine and thus inhibiting serine biosynthesis.

To validate the proteomics results, we used RT-qPCR to examine the expression of *glyA* and *trpA* in *napR_BCG_* OE. Consistent with the proteomics data, the relative mRNA expression levels of *glyA* and *trpA* were upregulated in *napR_BCG_* OE (Figure S8D).

In summary, our omics data indicate that NapR_BCG_ is a key regulator of intracellular amino acid metabolism in the *BCG vaccine*. Increased NapR_BCG_ expression orchestrates a coordinated modulation of four distinct serine metabolic pathways, ultimately leading to a reduction in intracellular serine levels.

## 3. Discussion

**Figure 7:**
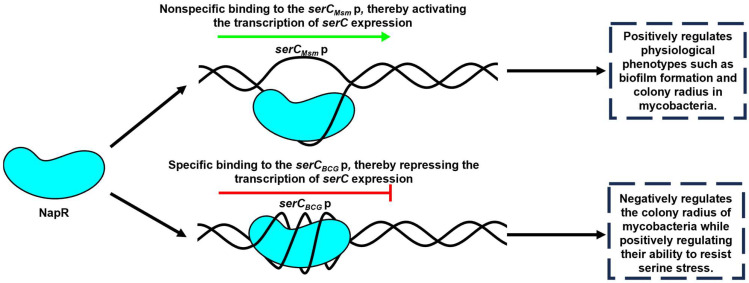
Schematic summary of the major findings of this study. NapR can act as either a transcriptional activator or repressor depending on the *serC* promoter variant, leading to distinct regulatory outcomes in pathogenic versus nonpathogenic mycobacterial strains.

In this study, we investigated the regulatory relationship between NapR and *serC*, and explored their impact on mycobacteria growth, colony morphology, and biofilm formation in both nonpathogenic strains, represented by *M. smegmatis,* and pathogenic strains, represented by the *BCG vaccine*. Our findings demonstrate that NapR functions as a transcriptional regulator of *serC* by binding directly to the *serC* promoter; however, the regulatory mechanism and its physiological consequences exhibit significant strain-specific differences. In *M. smegmatis*, NapR_Msm_ positively regulates colony radius and biofilm formation by activating *serC_Msm_*. In contrast, in the *BCG vaccine*, NapR_BCG_ exerts a negative regulatory effect on *BCG vaccine* colony size, but facilitates the *BCG vaccine*’s tolerance to serine stress by repressing *serC_BCG_* (Figure 7).

As the key enzyme in serine metabolism, SerC is essential for mycobacterial growth, with its repression leading to significant growth defects on solid media. Intriguingly, NapR activates *serC* in pathogenic strains but represses *serC* in nonpathogenic strains. The contrasting regulatory effects of NapR might be associated with the form and degree of its binding to the *serC* promoter. EMSA experiments revealed that at a concentration of 5 μM, both NapR_Msm_ and NapR_BCG_ could fully bind 50 ng of the *serC* promoter from the *BCG vaccine* (Figure 4A and Figure S9A), whereas neither protein was unable to completely bind 50 ng of the *serC* promoter from *M. smegmatis* under the same conditions (Figure 2A and Figure S9A). Despite the high degree of homology between NapR_Msm_ and NapR_BCG_, the sequence homology of the *serC* promoter regions in these two species is relatively low (Figure S9B). The *BCG vaccine* and *M. tuberculosis* share a high degree of homology. The homology between *serC_BCG_*p and *serC_Mtb_* p is 100% (Figure S9B), and the homology of NapR and SerC between *M. tuberculosis* and *BCG vaccine* is also 100% (Figure S10B). We conclude that NapR functions as a repressor of SerC in *M. tuberculosis*. Based on these findings, we speculate that variations in the *serC* promoter sequence leads to the strong and specific binding of NapR to the *serC* promoter in pathogenic strains, and, on the other hand, weak and nonspecific binding in nonpathogenic strains, likely contributing to the observed differences in the regulatory outcomes.

According to the widely accepted biofilm development model, biofilm formation can be summarized into reversible attachment, irreversible attachment, maturation, and dispersion [27]. The maturation phase can be further divided into three stages: the microcolony phase, the macrocolony phase, and the mature biofilm phase [18]. However, the precise distinctions between microcolonies and macrocolonies remain to be fully elucidated. A recent study in *Pseudomonas aeruginosa* demonstrated that Psl polysaccharides are evenly distributed throughout microcolonies, whereas in macrocolonies, Psl is predominantly localized at the biofilm periphery [28]. In our study, *serC* repression inhibited biofilm formation in *M. smegmatis* (Figure 2E,F). Further experiments showed that while *serC* repression does not affect growth in static liquid cultures, plating these cultures onto 7H10 solid media not only resulted in a colony count reduced to half that of the wild-type strain, but also inhibited the transition from microcolonies to macrocolonies (Figure 3B). Moreover, in a strain lacking *napR* with concomitant *serC* repression, the colony count was even reduced to approximately one-tenth that of the wild-type (Figure 3A and Figure S3B). This phenomenon is likely due to reduced attachment of *M. smegmatis* to 7H10 agar as a result of *serC* repression. The different effect in liquid and solid media can likely be attributed to nutrient availability and diffusion dynamics, as well as the cell–cell interactions and communication. Notably, this growth inhibition on solid medium could not be completely alleviated by serine supplementation, suggesting that the effects of *serC* repression on solid-phase growth extend beyond serine metabolism. Although *serC* is necessary for the serine biosynthesis pathway, it is also interconnected with other amino acid metabolic pathways. Therefore, serine supplementation alone could not restore the broader metabolic imbalances due to *serC* repression. Altogether, our study revealed that *serC* repression impairs biofilm formation in *M. smegmatis* through a multi-faceted mechanism, encompassing both defects in initial attachment to solid surfaces and an inhibition of the transition from microcolonies to macrocolonies during biofilm development.

Beyond its role in biosynthesis and cell growth, serine metabolism plays a pivotal role in eukaryotic anti-infection processes. Lipopolysaccharide (LPS) can directly stimulate Toll-like receptors on macrophages, thereby activating intracellular serine synthesis [29]. Through the provision of one-carbon units to the methionine cycle, serine promotes the production of S-adenosylmethionine (SAM), which, in turn, modifies histones or DNA via methylation and stimulates macrophages to release IL-1β [29]. IL-1β, a key pro-inflammatory cytokine, plays a crucial role in macrophage-mediated resistance to *M. tuberculosis*. It was reported that mice infected with *M. tuberculosis* and lacking IL-1β exhibit significantly reduced survival rate rates compared to wild-type mice [30]. IL-1β can also act on epithelial cells to produce antimicrobial peptides, such as DEFB4/HBD2, and promotes endothelial cell expression of adhesion molecules, facilitating the recruitment of other immune cells to sites of infection [31]. To counteract these host defense mechanisms, *M. tuberculosis* has evolved strategies to inhibit IL-1β secretion. For instance, Rv0198c, a *M. tuberculosis* protein, suppresses IL-1β secretion by inhibiting inflammasome activation [32]. As an environmental microbe frequently exposed to nutrient-poor conditions, *M. smegmatis* must acquire large amounts of serine from its surroundings to survive. NapR_Msm_, functioning as a transcriptional activator of *serC_Msm_*, facilitates serine acquisition in *M. smegmatis*. In contrast, *M. bovis*, a pathogenic bacterium that resides within macrophages, faces a different challenge. Within the macrophage environment, high serine levels may serve as an indicator of host immune activation, particularly IL-1β production. To counteract this potential threat, *M. bovis* may detect high serine levels and quickly shift into a metabolically repressed state to cope with the threat posed by macrophage activation. This inference could explain our findings that high concentrations of serine inhibit the growth of the *BCG vaccine* strain, a close relative of *M. bovis*, while exhibiting minimal impact on *M. smegmatis* growth (Figure S3A and Figure 5B), suggesting that the *BCG vaccine* has evolved a heightened sensitivity to serine-induced stress. Accordingly, overexpression of NapR_BCG_, by repressing *serC_BCG_* and orchestrating a coordinated modulation of other distinct serine metabolic pathways, results in a reduction in intracellular serine levels and then mitigates the detrimental effects of elevated serine levels within the macrophage environment. This mechanism enables the *BCG vaccine* strain to better withstand the host immune response (Figure 5B and Figure 6D).

Overall, our findings demonstrate that SerC is critical for mycobacteria, and its repression has profound impacts on *M. smegmatis*. Previous studies have successfully resolved the protein structure of SerC in *M. tuberculosis*, revealing only 23.6% homology with human phosphoserine aminotransferase. This significant structural divergence indicates that SerC could serve as a promising new drug target for tuberculosis treatments amenable to drug discovery efforts utilizing virtual screening and molecular dynamics simulations [33,34]. Our findings also highlight the contrasting regulatory effects of NapR on *serC* expression in pathogenic and nonpathogenic mycobacteria, suggesting a key role for NapR during human infection. Although *napR* knockout in the *BCG vaccine* did not produce dramatic phenotypic changes under in vitro conditions, the extensive involvement of NapR in in serine metabolism and multiple stress responses in the *BCG vaccine* implies that targeting NapR may offer a novel therapeutic strategy to combat drug-resistant *M. tuberculosis* and enhance host-mediated immune responses.

In conclusion, we showed that NapR functions as a transcriptional regulator of *serC* by binding directly to the *serC* promoter. In *M. smegmatis*, NapR activates *serC_Msm_* transcription, positively impacting biofilm formation, the transition from microcolonies to macrocolonies, and growth on solid media. In contrast, in the *BCG vaccine* strain, NapR functions as a transcriptional repressor of *serC_BCG_*, contributing to the *BCG vaccine*’s tolerance to serine stress. These findings highlight the contrasting regulatory roles of NapR in different mycobacterial species and underscore the importance of serine metabolism in bacterial adaptation and survival. This study provides valuable insights into the complex interplay between metabolism, gene regulation, and bacterial physiology, with potential implications for the development of novel anti-mycobacterial therapies.

## 4. Material and Methods

### 4.1. Cloning

Primers containing restriction sites were designed by selecting 20 bp sequences upstream and downstream of the target gene. The primers were synthesized by Tsingke Biotech (Beijing, China). The plasmid pMV261 was used to overexpress the target gene, and pET-cm1 was used for protein purification. PCR-amplified DNA fragments were ligated into the plasmids using T4 DNA ligase, and the ligated products were then chemically transformed into *E. coli* DH5α. The transformants were cultured in LB medium (20 g/L tryptone (OXOID, Basingstoke, UK), 10 g/L yeast extract (OXOID, Basingstoke, UK), 20 g/L NaCl (Sangon Biotech, Shanghai, China), pH 7.0), and the recombinant plasmids were extracted using the alkaline lysis method. To confirm successful construction, PCR amplification of the target gene from the recombinant plasmid was performed, and the size of the recombinant plasmid was compared with that of the original plasmid.

### 4.2. Protein Expression and Purification

The constructed pET-cm1 plasmid harboring the target gene was chemically transformed into *E. coli* BL21(DE3), followed by expansion in LB medium. The culture was incubated at 37 °C and 160 rpm until the OD_600_ reached 0.6. The temperature was then lowered to 16 °C, and isopropyl-β-D-1-thiogalactopyranoside (IPTG) (Solarbio, Beijing, China) was added to a final concentration of 0.4 mM. After 12 h of induction, cells were harvested by centrifugation, and the cell pellets were disrupted using a 300 W ultrasonic homogenizer. Cellular debris was collected by centrifugation at 8000× *g* rpm for 30 min. Ni-NTA (Solarbio, Beijing, China) agarose affinity chromatography was employed to purify proteins containing His tags: column washes were performed with 50 mM and 100 mM imidazole (Solarbio, Beijing, China), while the target protein was eluted using 250 mM imidazole. The eluted proteins were dialyzed against protein dialysis buffer (20 mM Tris-base (Sigma-Aldrich, Saint Louis, MO, USA), 100 mM NaCl, 10% *v*/*v* glycerol (Sangon Biotech, Shanghai, China)). Purified proteins were examined by sodium dodecyl sulfate–polyacrylamide gel electrophoresis (SDS–PAGE), and their concentrations were measured with a NanoDrop (Thermo Fisher Scientific, Waltham, MA, USA) instrument.

### 4.3. Electrophoretic Mobility Shift Assay

The promoter fragment of *serC* was obtained via PCR. An 8% native polyacrylamide (Solarbio, Beijing, China) gel was prepared. The promoter fragment was mixed with protein dialysis buffer to form a total volume of 17.5 μL containing various concentrations of protein, along with an additional 1.5 μL of H buffer (Takara, Dalian, China) and 1 μL of 50% (*v*/*v*) glycerol. The mixture was incubated for 15 min. Afterward, the incubated solution was loaded onto the previously prepared 8% polyacrylamide gel and subjected to electrophoresis at 150 V for 1 h in TBE buffer (5.4 g/L Tris-base, 2.75 g/L boric acid (Tianjin Aopusheng Chemical Co., Ltd., Tianjin, China), 0.372 g/L Na_2_EDTA·2H_2_O (Solarbio, Beijing, China), pH 8.3). The gel was then stained with ethidium bromide (EB) (Solarbio, Beijing, China), and images were captured using a GelDoc scanner (BIO-RAD, Hercules, CA, USA).

### 4.4. Construction of Target Gene Knockdown/Overexpression Strains

Knockdown plasmid construction: To knock down target gene expression, potential PAM sites on the full-length *serC* gene were identified, and complementary paired sgRNA primers targeting *serC* were designed. *Bsm* BI restriction sites were incorporated into the primers. After annealing, the double-stranded DNA was inserted into either the pLJR 962 vector (for *M. smegmatis*) or the pLJR 965 vector (for the *BCG vaccine*) through restriction digestion and ligation, thereby creating the corresponding recombinant plasmids.

Overexpression plasmid construction: For overexpression, *napR* was cloned into the pMV261+ vector to generate the recombinant plasmid. A total of 200 ng of the recombinant plasmid was introduced into competent *M. smegmatis* or *BCG vaccine* cells. Electroporation was performed at 2500 V, after which the cells were allowed to recover in 7H9 liquid medium (4.9 g/L Middlebrook 7H9 Broth (BD Difco, Franklin Lakes, NJ, USA), 0.2% *v*/*v* glycerol, 0.05% *v*/*v* Tween-80). The *M. smegmatis* cultures were recovered for 4 h, and the *BCG vaccine* cultures were recovered for 24 h. The recovered cells were then plated on 7H10 solid medium (19 g/L Middlebrook 7H10 Powder (BD Difco, Franklin Lakes, NJ, USA), 0.2% *v*/*v* glycerol, 0.03 g/L kanamycin (Solarbio, Beijing, China)) and incubated—3–4 days for *M. smegmatis* and 25–30 days for the *BCG vaccine*. Primers designed to validate the presence of the recombinant plasmid were used in PCR with bacterial lysates (prepared by heating to 100 °C for 10 min), and the resulting amplicons were analyzed to confirm the successful construction of the recombinant strains.

### 4.5. Real-Time Quantitative PCR (RT-qPCR)

When the constructed *M. smegmatis*/*BCG vaccine* cultures reached an OD_600_ of 1.0, the cells were harvested and washed twice with PBS. The cell pellets were then resuspended in 200 μL of 3 mg/mL lysozyme (Solarbio, Beijing, China) and incubated at 37 °C for 30 min. Total bacterial RNA was extracted using an RNA extraction kit from Aidlab (Beijing, China). A 20 μL aliquot of the extracted RNA was treated with 1 μL of DNase I (Takara, Dalian, China) and 2 μL of 10× DNase I buffer (Takara, Dalian, China) at 37 °C for 30 min to remove any residual genomic DNA. The reaction mixture was then heated at 65 °C for 10 min to inactivate DNase I. Using a reverse transcription kit, mRNA was reverse-transcribed. The concentration of total RNA was measured with a NanoDrop, and the reaction volume for reverse transcription was set to 40 μL, which included 300 ng RNA, 2 ng random primer (Aidlab, Beijing, China), and 8 ng 5× TURE R Mix (Aidlab, Beijing, China).

Primers for qPCR were designed using *sigA* as the internal reference gene, where *msmeg_2758* served as *sigA* for *M. smegmatis*, and *bcg_2716* served as *sigA* for the *BCG vaccine*. Real-time quantitative PCR was performed using SYBR qPCR Green Master Mix (Aidlab, Beijing, China). The relative expression of the target gene was calculated as 2^−ΔΔCt^.

### 4.6. Observation of Colony Surface Morphology

Activated mycobacterial cultures were grown in 7H9 medium containing kanamycin until OD_600_ reached 1.0. Subsequently, 7H10 agar plates with kanamycin—and, where applicable, 0.1 ng/mL anhydrotetracycline (Solarbio, Beijing, China)—were prepared. Two microliters of the OD_600_ = 1.0 culture were spotted onto the surface of 7H10 agar plates (with or without anhydrotetracycline). *M. smegmatis* plates were incubated statically at 37 °C for 3–5 days, while *BCG vaccine* plates were incubated at 37 °C for 20–30 days. Colony size and surface wrinkling were then observed.

### 4.7. Biofilm Assays

Activated mycobacterial cultures were grown in 7H9 medium containing kanamycin to OD_600_ = 1.0. The cells were harvested and resuspended in M63 salt medium (13.6 g/L KH_2_PO_4_ (Solarbio, Beijing, China), 4.2 g/L KOH (Tianjin Beichen Fangzheng Reagent Factory, Tianjin, China), 1.98 g/L (NH_4_)_2_SO_4_ (Sinopharm Chemical Reagent Co., Ltd., Shanghai, China), 1.08 mg/L FeSO_4_ (Tianjin New Technology Industrial Park Kemao Chemical Reagent Co., Ltd. Tianjin, China), 50 mL 40% (*v*/*v*) glucose (Tianjin AoboKai Chemical Co., Ltd., Tianjin, China), 10 mL 20% (*v*/*v*) casein hydrolysate (Macklin, Shanghai, China), 10 mL 100 mM MgSO_4_ (Sinopharm Chemical Reagent Co., Ltd., Shanghai, China), 7 mL 100 mM CaCl_2_ (Tianjin Aopusheng Chemical Co., Ltd., Tianjin, China), 0.03 g/L kanamycin). The resuspended cells were then diluted in M63 medium to OD_600_ = 0.3, and the resulting cell suspension was divided into two portions—one supplemented with anhydrotetracycline to a final concentration of 0.1 ng/mL, and the other without anhydrotetracycline. Three milliliters of each suspension were dispensed into the wells of a 12-well plate.

Separately, the OD_600_ = 0.3 suspension was further diluted to OD_600_ = 0.1, divided into two portions (with or without anhydrotetracycline), and 100 μL of each was dispensed into the wells of a 96-well plate.

Air–Liquid Interface Biofilm Assay: The 12-well plate was incubated statically at 30 °C for 3–5 days. Biofilm formation was monitored daily, and images were taken to record changes.

Biofilm Quantification Assay: The 96-well plate was incubated at 37 °C and 80 rpm for 36–48 h. Biofilms were then stained with 120 μL of 1% crystal violet (Solarbio, Beijing, China) solution for 30 min, followed by rinsing with deionized water to remove excess dye. The remaining crystal violet bound to the biofilm was dissolved in an ethanol–acetone solution (20% (*v*/*v*) ethanol (Sangon Biotech, Shanghai, China), 80% (*v*/*v*) acetone (Sigma-Aldrich, Saint Louis, MO, USA)) for 5 min, and the absorbance at 570 nm (OD_570_) was measured with a microplate reader.

### 4.8. Growth Curve Assays

Activated mycobacterial cultures were grown in 7H9 medium containing kanamycin to OD_600_ = 1.0. The cells were collected, resuspended in fresh 7H9 medium, and transferred to 100 mL 7H9 medium containing kanamycin at a starting OD_600_ = 0.15. If CRISPRi strains were used, anhydrotetracycline was added to a final concentration of 0.1 ng/mL. For *M. smegmatis*, samples (2 mL) were taken every 4 h to measure OD_600_; for the *BCG vaccine*, samples were collected every 24 h. The measured OD_600_ values were subsequently converted to CFU counts.

### 4.9. Colony Counting Assays

Activated mycobacterial cultures were grown in 7H9 medium containing kanamycin to OD_600_ = 1.0. The cells were then harvested and resuspended in fresh 7H9 medium. The resuspended cells were inoculated into 100 mL of 7H9 medium containing kanamycin and 0.1 ng/mL anhydrotetracycline, with a starting OD_600_ = 0.6. Cultures were incubated statically at 37 °C for 48 h, and OD_600_ was recorded. The culture was then diluted 1:20,000, and 100 μL of the diluted suspension was plated onto 7H10 agar containing kanamycin. Plates were incubated statically at 37 °C for 3–5 days (for *M. smegmatis*) or 20–30 days (for the *BCG vaccine*), after which the number of colonies on the 7H10 plates were counted.

### 4.10. Proteomic Analysis

Activated *BCG vaccine* cultures were grown in 7H9 medium containing kanamycin until the OD_600_ reached 1.2. The cells were harvested, washed with PBS, and frozen in liquid nitrogen. Protein extraction, enzymatic digestion, and liquid chromatography–tandem mass spectrometry (LC-MS/MS) analysis were performed by Hangzhou Jingjie Biotechnology Co., Ltd. (Hangzhou, China). The bacterial pellets were then lysed in a buffer consisting of 8 M urea (Sigma-Aldrich, Saint Louis, MO, USA) and 1% protease (Merck Millipore, Billerica, MA, USA) inhibitor cocktail using a high-intensity ultrasonic processor (Scientz, Ningbo, China), followed by centrifugation to remove cell debris. Next, the proteins were reduced with 5 mM dithiothreitol (DTT) (Sigma-Aldrich, Saint Louis, MO, USA) at 56 °C for 30 min, then alkylated with 11 mM iodoacetamide (Sigma-Aldrich, Saint Louis, MO, USA) in the dark at room temperature for 15 min. The protein sample was diluted with 100 mM TEAB (Sigma-Aldrich, Saint Louis, MO, USA) until the urea concentration was below 2 M. Finally, trypsin (Promega, Madison, WI, USA) digestion was performed overnight at a trypsin-to-protein mass ratio of 1:50, followed by a second digestion for 4 h at 1:100. The resulting peptides were desalted using a C_18_ SPE column (ThermoFisher Scientific, Waltham, MA, USA).

After being dissolved in Liquid Phase A (0.1% formic acid (Sigma-Aldrich, Saint Louis, MO, USA), 2% acetonitrile (Thermo Fisher Scientific, Waltham, MA, USA), 98% deionized water), the peptides were separated on a NanoElute ultra-high-performance liquid chromatography (UHPLC) system (Bruker Corporation, Billerica, MA, USA). Mobile Phase A was 0.1% formic acid, 2% acetonitrile, and 98% deionized water; Mobile Phase B was 0.1% formic acid in 100% acetonitrile. The gradient was set as follows: 0–40 min, 6–24% B; 40–52 min, 24–35% B; 52–56 min, 35–80% B; 56–60 min, 80% B, at a flow rate of 450 nL/min. The separated peptides were then introduced into a capillary ion source for electrospray ionization and analyzed using a timsTOF Pro 2 mass spectrometer (Bruker Corporation, Billerica, MA, USA). The ion source voltage was set at 1.65 kV, and both the precursor ions and their corresponding fragments were detected at high resolution by the TOF analyzer. The MS/MS scan range was 100–1700 *m*/*z*. Data were acquired in parallel accumulation–serial fragmentation (PASEF) mode: after each full MS scan, 10 PASEF MS/MS scans were triggered for precursor ions with charges ranging from 0 to 5. The dynamic exclusion time was set to 30 s to avoid repeated scans of the same precursor ions.

The raw mass spectrometry data were imported into a database search engine, with the relevant analytical parameters set according to the experimental design. In this experiment, the MS2 data were processed using MaxQuant (v1.6.15.0) against the Blast_Mycobacterium_bovis_strain_BCG_410289_PR_20220526.fasta database. A reverse database was added to calculate the false discovery rate (FDR) caused by random matches, and a common contaminant database was included to eliminate the influence of contamination. The enzyme specificity was set to Trypsin/P, with up to two missed cleavage sites allowed. The minimum peptide length was set to seven amino acid residues, and a maximum of five modifications per peptide was allowed. The mass tolerances for both the first (First search) and main (Main search) searches were 20 ppm, and the MS/MS tolerance was also 20 ppm. Carbamidomethylation of cysteine (C) was set as a fixed modification, while methionine oxidation and protein N-terminal acetylation were set as variable modifications. The FDR for both protein and PSM identifications was set to 1%.

GO and KEGG enrichment analyses of the proteomic data were performed using the R language (v4.2.2) package “clusterProfiler” (v4.6.2). Volcano plots and KEGG enrichment diagrams were generated using the R package “ggplot2” (v3.5.0), while GO enrichment results were visualized with “circlize” (v0.4.16), and heatmaps were plotted using “pheatmap” (v1.0.12). Protein–protein interaction analyses were conducted via the STRING database, and protein interaction networks were visualized with the R package “igraph” (v1.4.1).

### 4.11. Statistical Analysis

All data are presented as the mean ± S.E.M. and represent results from at least three independent experiments. Two-tailed Student’s *t*-tests with equal variance assumptions were performed using Microsoft Excel (v2408, Build 16.0.17932.20252), and *p*-value < 0.05 was considered statistically significant.

## Data Availability

The original contributions presented in this study are included in the article. Further inquiries can be directed to the corresponding author. The proteomics data of the *BCG vaccine* have been deposited in the Proteomics Identification Database (ID: PXD059682).

## Supplementary Materials

The following supporting information can be downloaded at: https://www.mdpi.com/article/10.3390/ijms26052181/s1.
