# Supplementary material for: NapR Regulates the Expression of Phosphoserine Aminotransferase SerC to Modulate Biofilm Formation and Resistance to Serine Stress of Mycobacteria
Source: Int J Mol Sci. 2025 Feb 28;26(5):2181. doi: 10.3390/ijms26052181 (PMC11899882; doi:10.3390/ijms26052181)
Supplement: Supplementary file 1 [file ijms-26-02181-s001.zip › ijms-3459354-supplementary.pdf]

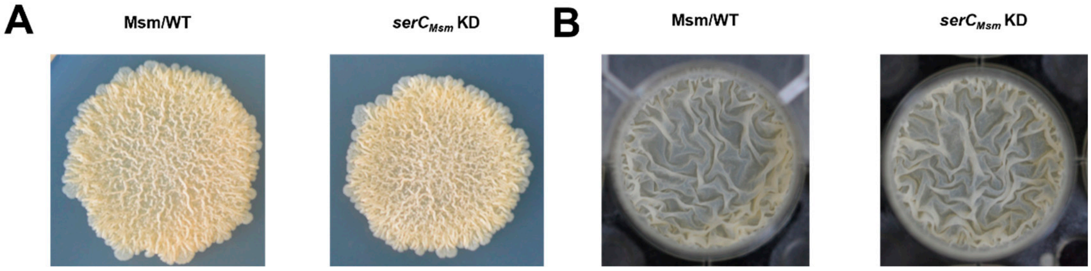

Supplementary Figure S1. Effects of *serC*$_{Msm}$ repression on *M. smegmatis* phenotypes. (**A**) The colony morphology of Msm/WT and *serC*$_{Msm}$ KD strains in the absence of anhydrotetracycline. (**B**) The biofilm formation of Msm/WT and *serC*$_{Msm}$ KD strains in the absence of anhydrotetracycline.

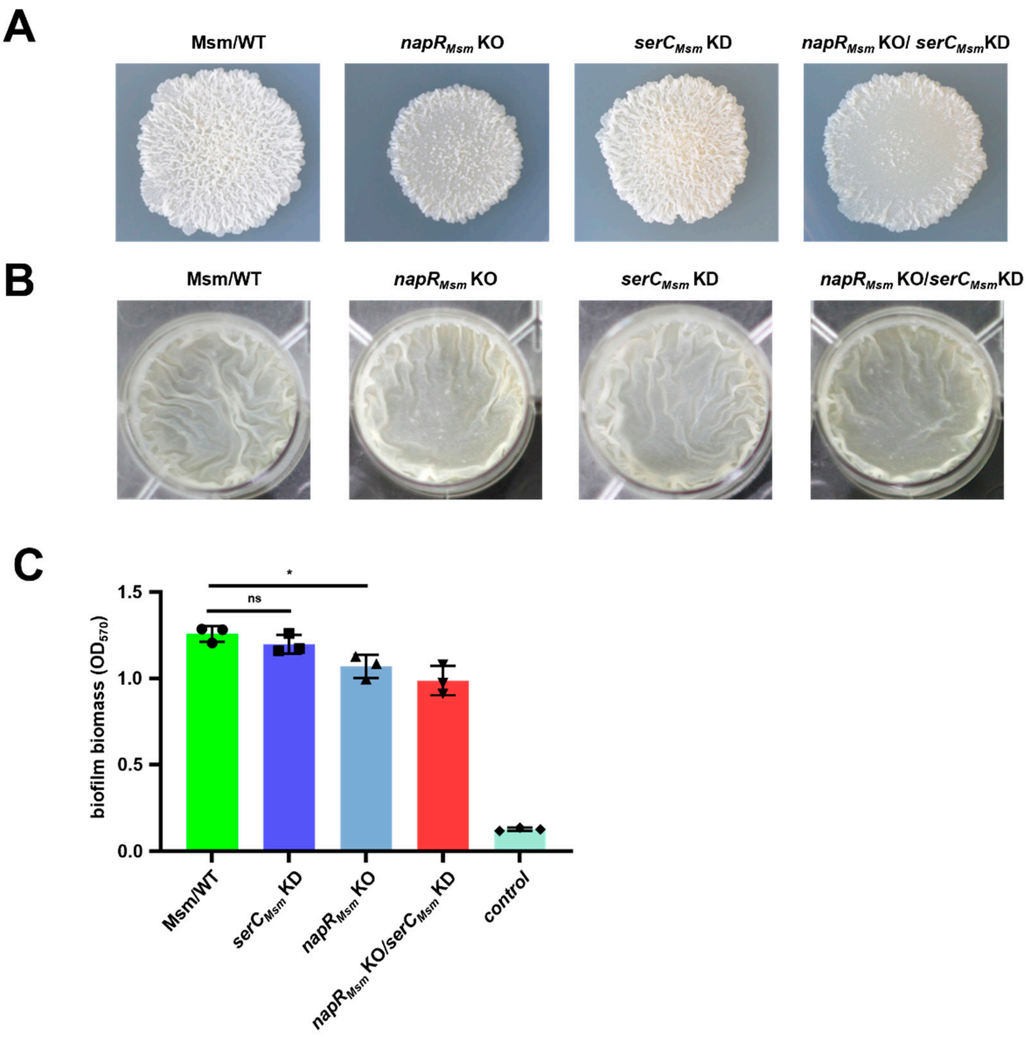

Supplementary Figure S2. Effects of *serC*$_{Msm}$ repression or *napR*$_{Msm}$ depletion on *M. smegmatis* phenotypes. (**A**) Spot colony morphology of Msm/WT, *napR*$_{Msm}$ KO, *serC*$_{Msm}$ KD and *napR*$_{Msm}$ KO/*serC*$_{Msm}$ KD strains in the absence of anhydrotetracycline. (**B**) The biofilm formation at the air-liquid surface of Msm/WT, *napR*$_{Msm}$ KO, *serC*$_{Msm}$ KD and *napR*$_{Msm}$ KO/*serC*$_{Msm}$ KD strains in the absence of anhydrotetracycline. (**C**) Quantitation of biofilm biomass by crystal violet staining in the absence of anhydrotetracycline. ns $P \geq 0.05$, *$P < 0.05$.

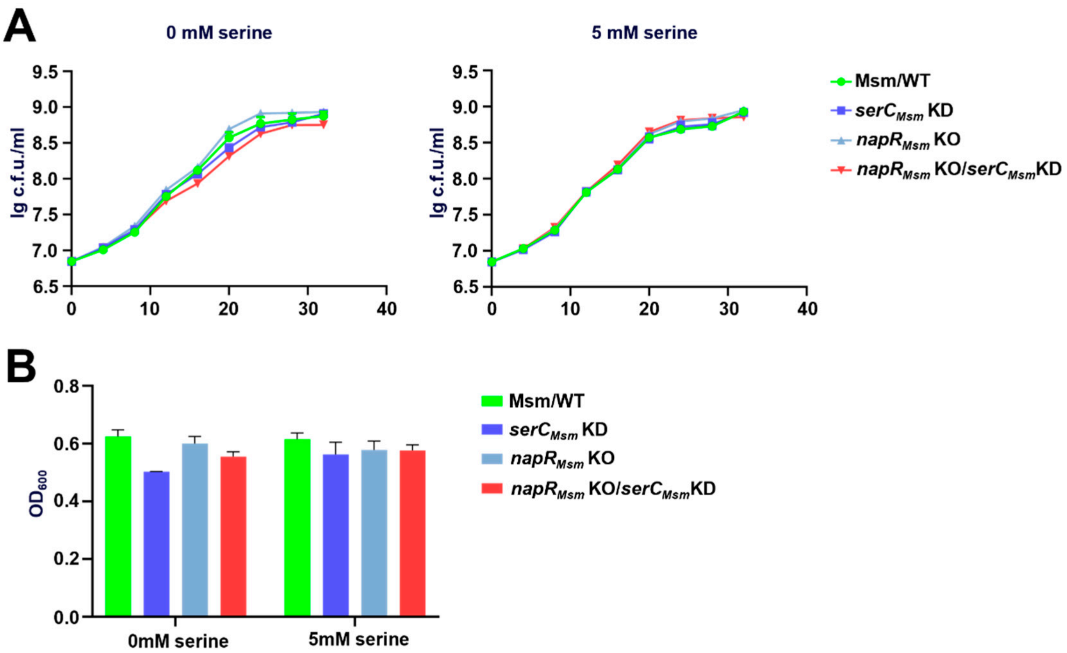

Supplementary Figure S3. *serC_{Msm}* repression or *napR_{Msm}* knockout causes mild growth inhibition of *M. smegmatis* in liquid culture. (A) Growth curves of *M. smegmatis* strains under shaking conditions at 37°C and 160 rpm. (B) Growth assessment of *M. smegmatis* strains after 48 hours of static incubation at 37°C.

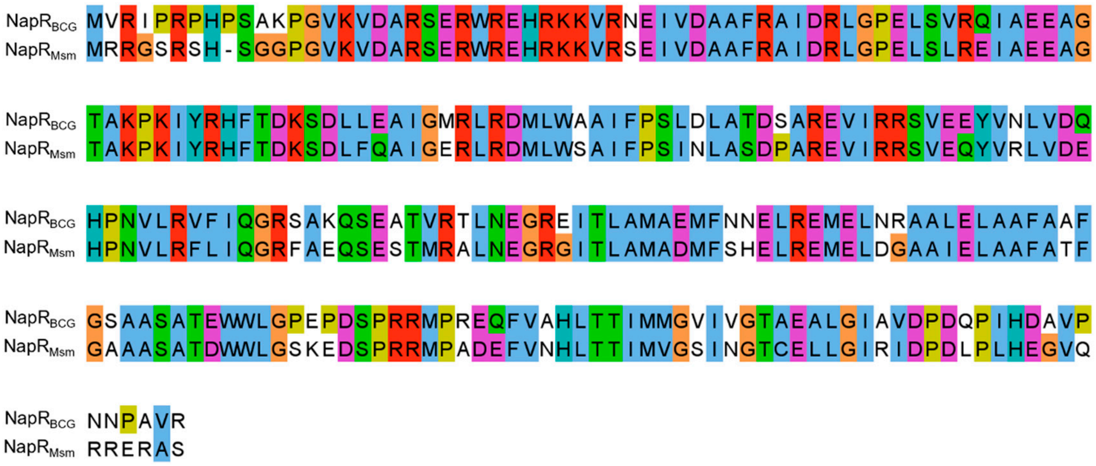

Supplementary Figure S4. Sequence alignment of $NapR_{BCG}$ and $NapR_{Msm}$.

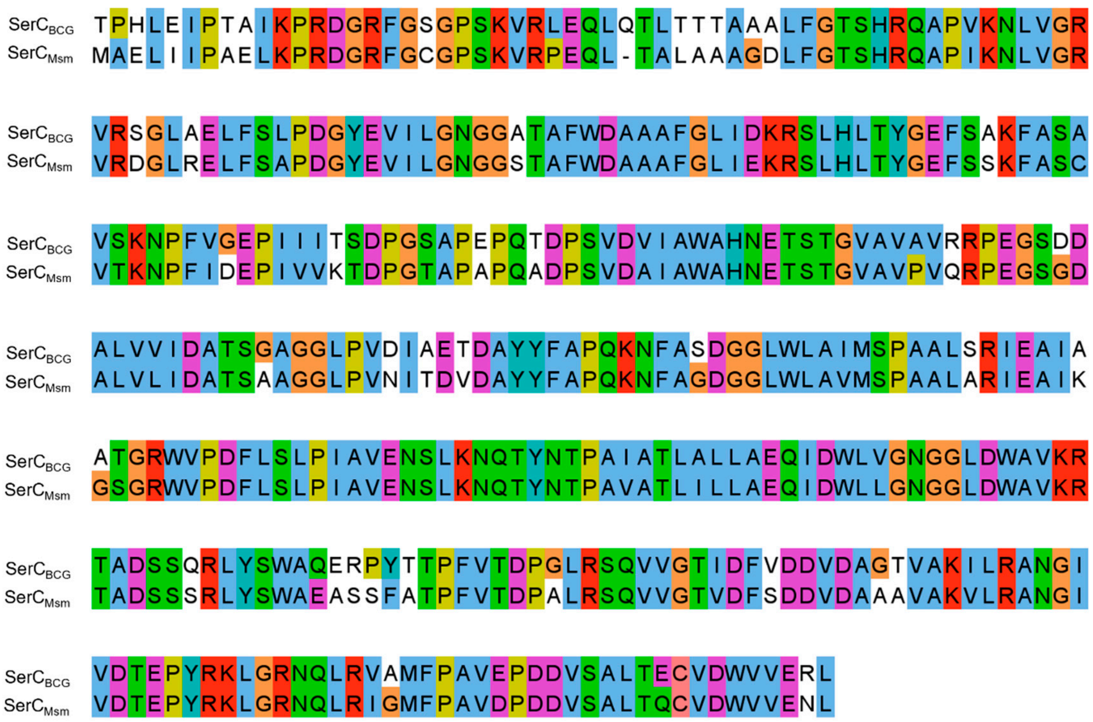

Supplementary Figure S5. Sequence alignment of $SerC_{BCG}$ and $SerC_{Msm}$.

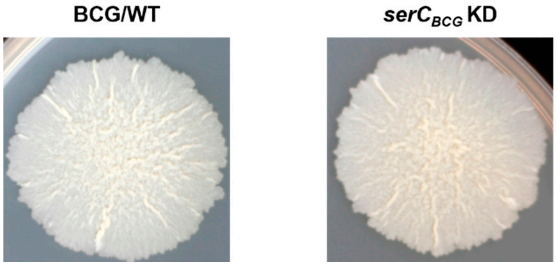

Supplementary Figure S6. The colony morphology of BCG/WT and *serC*$_{BCG}$ KD strains in the absence of anhydrotetracycline.

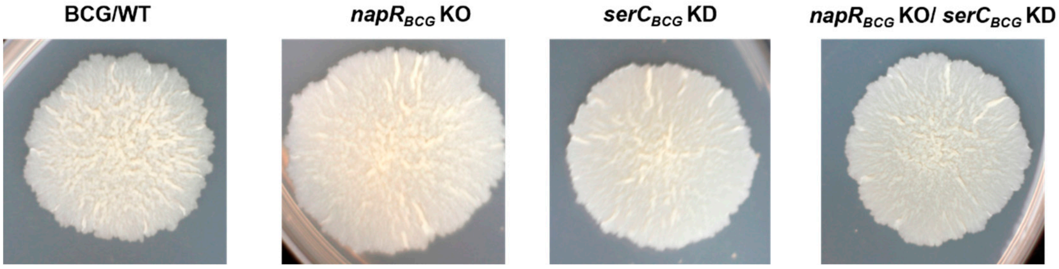

Supplementary Figure S7. The colony morphology of BCG/WT, *napR*$_{BCG}$ KO, *serC*$_{BCG}$ KD and *napR*$_{BCG}$ KO/*serC*$_{BCG}$ KD strains in the absence of anhydrotetracycline.

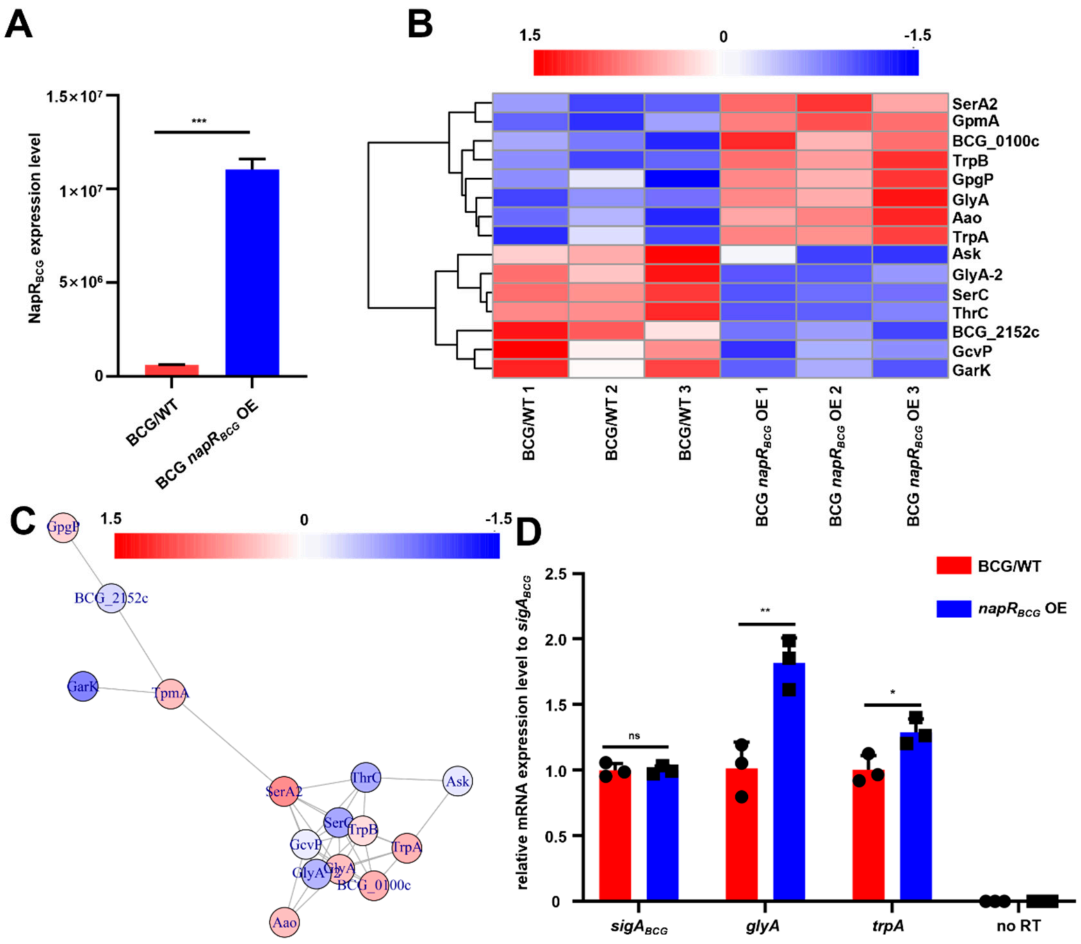

Supplementary Figure S8. Proteomic analysis for the targets of *napR*$_{BCG}$. (**A**) Proteomic data confirming successful overexpression of *napR*$_{BCG}$. (**B**) Heatmap of enriched proteins in the glycine, serine, and threonine metabolism pathway. (**C**) Protein–protein interaction (PPI) network analysis of the enriched proteins in the glycine, serine, and threonine metabolism pathway. (**D**) RT-qPCR analysis for the expression of levels of *trpA* and *glyA* in BCG/WT and *napR*$_{BCG}$ KO strains. *sigA*$_{BCG}$ was as a reference control. ns $P \geq 0.05$, *$P < 0.05$, **$P < 0.01$, ***$P < 0.001$.

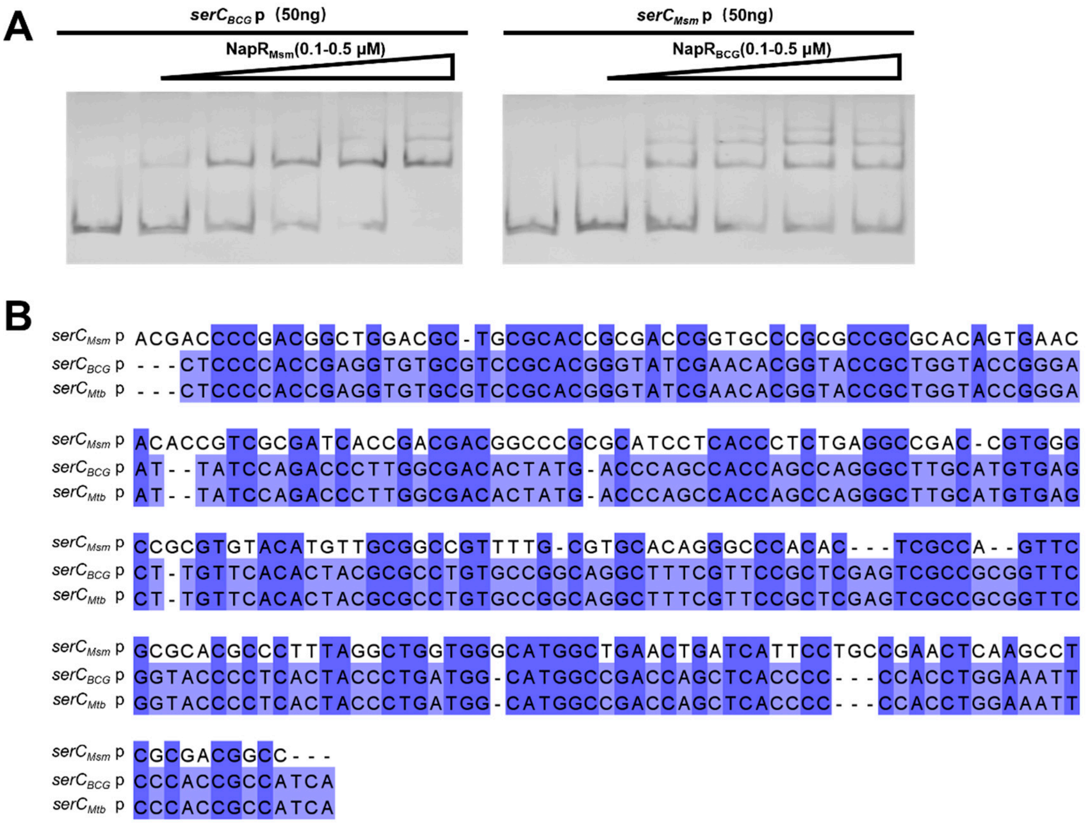

Supplementary Figure S9. Comparative analysis of the *serC*$_{Msm}$ and *serC*$_{BCG}$ promoters. (**A**) EMSA assays for the *serC*$_{BCG}$ promoter DNA-binding activity of NapR$_{Msm}$, and the *serC*$_{Msm}$ promoter DNA-binding activity of NapR$_{BCG}$. (**B**) Sequence alignment of the *serC*$_{Msm}$ p, *serC*$_{BCG}$ p and *serC*$_{Mtb}$ p regions.

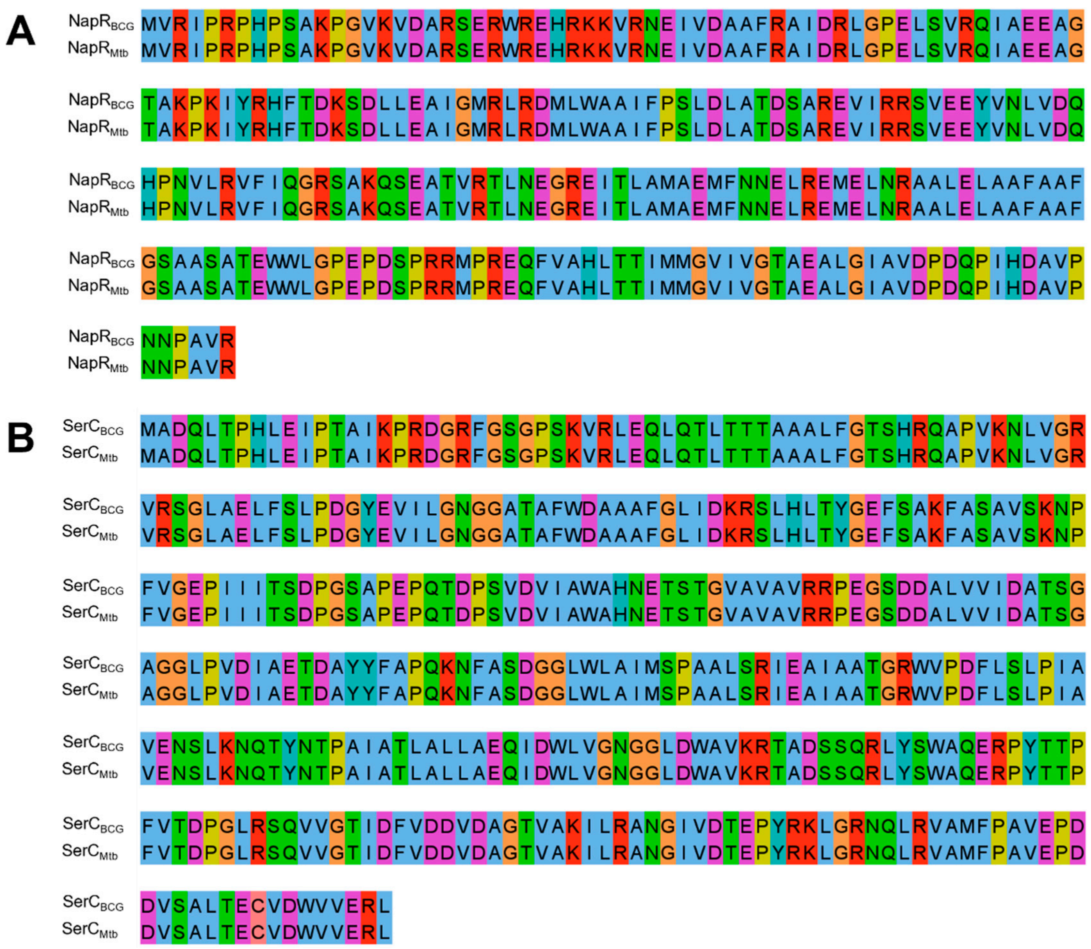

Supplementary Figure S10. Sequence alignment of NapR and SerC in *BCG Vaccine* and *Mycobacterium tuberculosis*. (**A**) Sequence alignment of $NapR_{BCG}$ and $NapR_{Mtb}$. (**B**) Sequence alignment of $SerC_{BCG}$ and $SerC_{Mtb}$.
